# Characteristics, therapy, and outcome of rare functioning pancreatic neuroendocrine neoplasms

**DOI:** 10.1038/s41598-024-68290-1

**Published:** 2024-08-09

**Authors:** Max B. Albers, Martina Sevcik, Dominik Wiese, Jerena Manoharan, Anja Rinke, Moritz Jesinghaus, Detlef K. Bartsch

**Affiliations:** 1 Department of Visceral, Thoracic and Vascular Surgery, University Hospital Marburg, Philipps University Marburg, Marburg, Germany; 2 Department of Internal Medicine, Division of Gastroenterology and Endocrinology, University Hospital Marburg, Philipps University Marburg, Marburg, Germany; 3 Institute of Pathology, University Hospital Marburg, Philipps University Marburg, Marburg, Germany

**Keywords:** Glucagonoma, VIPoma, Calcitoninoma, Pancreatic neuroendocrine neoplasia, Endocrinology, Gastroenterology, Oncology

## Abstract

Functioning pancreatic neuroendocrine neoplasms other than insulinomas and gastrinomas (rf-pNENs) are exceptionally rare tumours. Thus, their characteristics and long-term prognosis have not been well defined. This article aims to present data and experience from a single institution concerning this topic. Twelve of 216 (5.5%) patients with pNENs operated between 2002 and 2022 in the ENETS Centre of Excellence Marburg had rf-pNENs and their data were retrospectively analysed. We identified three vasoactive intestinal polypeptide producing pNENs, four glucagonomas and five calcitoninomas. The tumour could be visualised by preoperative imaging in all 12 patients, and six patients had distant metastases at the time of diagnosis. The tumour was located in the pancreatic tail in nine patients and the median tumour size was 82 (range 12–220) mm. Eleven patients underwent tumour resections (two robotic, nine conventional), nine of which were R0. After a median follow-up of 75 (range 1–247) months, six patients were alive, five of whom had no evidence of disease. All patients who remained disease-free had an initial R0 resection of the primary tumour and no initial liver involvement. This study sheds light on the distinct characteristics and outcomes of these exceedingly rare tumours, offering insights for improved understanding and management.

## Introduction

Rare functioning pancreatic neoplasms (rf-pNENs) comprise glucagonomas, vasoactive intestinal polypeptide (VIP) producing pNENs (VIPomas), calcitoninomas, somatostatinomas, as well as pNENs secreting parathyroid hormone related peptide, gonadotropin relasing hormone , adrenocorticotropic hormon, renin, luteinising hormone, erythropoietin and insulin-like growth factor II^1,2^. Rf-pNENs are extremely rare and account for only approximately 5% of functioning pNENs, with an annual incidence of approximately 0.05–0.1/1,000000/year^2–5^. The most frequent familial condition associated with rf-pNENs is multiple endocrine neoplasia type 1 (MEN1), with glucagonomas occurring in 5% and VIPomas in 3% of MEN1 patients^2,6^. Glucagonomas in the setting of MEN1 usually do not cause a syndrome, although glucagon serum levels may be significantly elevated. The diagnosis of an rf-pNEN requires at least the demonstration of an inappropriate elevation of the specific serum hormone (i.e. VIP or glucagon), ideally combined with symptoms caused by over-secretion of the distinct hormone. The diagnosis of rf-pNENs cannot be based solely on positive immunohistochemical results of resected specimens^2,5^, as these are frequently found also in tumours of asymptomatic patients without elevated hormone levels. As of 2022, approximately 600 glucagonomas^7–10^, 100 VIPomas^11–13^ and 60 calcitoninomas^14,15^ have been reported in case reports or small case series, whereas reports on other rf-pNENs do not exceed 10–25 cases per subtype^1,16–20^.

Glucagonoma, first described in 1942 by Becker^21^, is a pancreatic alpha-cell tumour secreting glucagon. It causes glucagonoma syndrome, which includes necrolytic migratory erythema, diabetes, venous thromboembolism, diarrhoea, stomatitis, and anaemia. However, in some series, only half of the patients had necrolytic migratory erythema, and only 20% developed diabetes before the diagnosis^10,22^. Thus, published cohorts in the literature comprise a heterogenous group of patients suffering from all named features of the syndrome, isolated symtpoms, and sometimes also include asymptomatic patients with glucagon expressing tumors in immunostaining.

VIPomas were first reported by Verner and Morrison in 1958^23^ and are characterised by excessive secretion of VIP, which may cause typical watery diarrhoea, hypokalaemia, and achlorhydria, the so-called WDHA syndrome.

Calcitonin-producing pNENs represent an extremely rare cause of hypercalcitoninemia, which is usually highly suggestive of medullary thyroid carcinoma^24^. Hormonal excess of calcitoninomas may cause diarrhoea and abdominal pain. Calcitonin producing pNENs appear to have a unique molecular signature compared to other pNEN subtypes^15^. A recent pathological study found that approximately 10% of all pNENs show a positive calcitonin immunoreactivity, whereas the corresponding serum calcitonin levels are rarely elevated^14^.

The average age at diagnosis of rf-pNENs is between 50 and 60 years with an equal gender distribution. A significant number of patients with rf-pNENs present with metastatic disease (40–80%) in the liver at initial diagnosis. The management of rf-pNENs is challenging because high-level evidence recommendations are currently lacking, especially for the treatment of symptomatic patients with diffuse metastatic tumours^5^. Surgical resection of all lesions is considered the only curative option. Symptom and tumour growth control can be achieved either with somatostatin analogues (SSAs), chemotherapy, and targeted therapy with sunitinib or peptide receptor radionucleotide therapy (PRRT)^5^. Debulking surgery and/or other cytoreductive techniques such as liver transarterial (chemo)embolisation (TACE) or radioembolisation can also be employed^5,16^. Nevertheless, because of the rarity of these tumours, experiences with more novel and specific therapeutic tools such as PRRT are still sparse. We present a series of 12 patients with rf-pNENs from a tertiary referral centre who were treated for 20 years. Clinicopathological characteristics, therapeutic modalities, and prognosis were evaluated.

## Patients and methods

### Patient cohort

Patients diagnosed with rf-pNENs were identified from the prospective pancreatic database of the Department of Visceral, Thoracic, and Vascular Surgery, Philipps-University Marburg, which was established in 2002 as a prerequisite for certification as an ENETS Centre of Excellence.

Patients with rf-pNENs were retrieved from this database and their clinical data were retrospectively analysed with special regard to demographics, clinical features, preoperative imaging, operative procedures, pathologic findings, and long-term follow-up.

Selective clinical data from our patients with calcitonin-producing pNENs have been reported previously^15,25^.

### Definition and diagnostics of Rf-pNENs

Rf-pNENs were defined according to the ENETS guidelines, such as VIPoma, glucagonoma, or calcitoninomas^5^. The diagnosis of a distinct rf-pNEN was confirmed if the pNEN was associated with at least a two-fold elevated serum hormone level and positive immunostaining for the respective hormone.

The fact that initially elevated serum hormone levels dropped to normal levels after potentially curative surgery was considered evidence that the resected pNEN was the source of hormone hypersecretion.

Preoperative imaging routinely includes abdominal ultrasonography, multidetector computed tomography (CT), and/or magnetic resonance imaging (MRI) with gadolinium, and endoscopic ultrasonography (EUS) if the tumor was limited to the pancreas and regional lymph nodes. Some patients also underwent somatostatin receptor scintigraphy (SRS) until 2013, which was then replaced by Gallium-68-positron-emission-tomography combined with CT (Ga68-DOTATOC PET/CT). In the case of resectable disease, and if fit for surgery, patients underwent pancreatic resection with or without metastasectomy. In cases of diffuse metastatic disease without complications (e.g., bowel obstruction), palliative debulking surgery was performed after a multidisciplinary tumour board decision. After potentially curative resection, no adjuvant treatment was administered, in accordance with ENETS guidelines^5,26^.

Tumours were classified according to the World Health Organization (WHO) classification 2017^27^ and defined as malignant^28^. All tumours were immunostained for Ki67, synaptophysin and chromogranin A. Ki67 ratio was determined as the percentage of positively stained tumour cells among the total number of malignant cells assessed. Potentially secreted hormones, such as VIP, glucagon, and calcitonin were verified by immunohistochemistry as described in previous publications^15,29^. Immunohistochemistry for synaptophysin, chromogranin A, and Ki67 was re-evaluated by an experienced pathologist (M.J.) at the time of this study.

### Perioperative complications

Complications of surgery were classified according to Clavien-Dindo^30^. Clinically relevant postoperative pancreatic fistula types B and C were defined according to the International Study Group of Pancreatic Fistula^31^. The length of hospital stay was not evaluated because several institutional changes in patient demission management over the years would have induced a significant bias.

### Follow-up

Follow-up was calculated from the time of the initial surgery to the time of last follow-up or death until the evaluation date of July 31, 2023. Disease-free survival was defined as the time from potentially curative surgery to disease recurrence, last follow-up without evidence of disease recurrence, or death.

An at least annual clinical follow-up was performed at our hospital or an associated physician with a laboratory workup, including the preoperatively increased specific hormone level, MRI of the abdomen, and in case of suspicion of recurrence or metastatic disease, additional somatostatin receptor imaging. Patients were classified as having no evidence of disease if there was no suspicion of symptom recurrence, the respective serum hormone levels were not elevated, and there were no visible tumours on imaging.

In the case of palliative surgical procedures and/or unresectable disease, patients were treated with a variety of modalities such as somatostatin analogues, PRRT, transarterial chemoembolisation, or chemotherapy, during follow-up upon recommendation of the multidisciplinary tumour board.

### Ethical standards

All patients provided written informed consent to register their data. While the treatment was conducted as part of routine clinical care, additional ethical approval was obtained for the retrospective analysis during this study from the local ethics committee of the University of Marburg (no. RS 22-51). All research was performed in accordance with relevant guidelines/regulations and in accordance with the Declaration of Helsinki.

### Statistics

Descriptive statistics were performed whenever applicable. Due to the small cohort size, analytical statistics were not reasonable.

## Results

In the study period from January 2002 to December 2022, 216 patients with pNENs underwent surgery at the ENETS Centre of Excellence Marburg. They included 156 patients with sporadic and 60 with MEN1-associated pNENs. Overall, 79 (36.6%) patients had functioning tumours, of which 12 (12/216, 5.5%) had rf-pNENs according to the actual ENETS definition^5^.

The demographic characteristics and symptoms of the 12 patients are summarised in Table 1. Five patients had a calcitoninoma, four patients had a glucagonoma and three patients had a VIPoma. Except for one patient with MEN1-associated VIPoma, the other 11 tumours were sporadic. Five patients were female. The median age at the time of surgery was 60 years (range 28–73 years). All 12 patients had at least two-fold elevated serum levels of the specific tumour-released hormone (median 31-fold, range 2–189-fold). CgA was measured preoperatively in ten patients and was elevated in eight cases (median 707ng/ml, range 211 – 15,174). All but three patients with calcitoninomas had specific symptoms. These three patients with calcitoninomas had unspecific symptoms (abdominal pain, weight loss) in combination with hypercalcemia, which where cured after surgery and therefore considered to be linked to the hormonal excess. Six patients had all above described features of a hormone-associated syndrome (three VIPomas, two glucagonomas, and one calcitonin producing pNEN). Diarrhoea was the most common symptom, presenting in six of 12 patients (three VIPomas, one calcitoninoma, and two glucagonomas).

**Table 1:** Demographic data and laboratory values.

Patient No	Gender	Age at Dx	Age at initial surgery	Symptoms before surgery	Tumor localisation	Serum hormone elevated	Serum hormone level before first surgery elevated (x-fold)^§^	CgA level before first surgery elevated (x-fold)^§^
1*	M	31	32	WDHA, abdominal pain	tail	VIP	10-fold	NA
2	F	61	73	WDHA	tail	VIP	2-fold	2.6-fold
3	M	60	70	WDHA, weight loss	tail	VIP	2.5-fold	21.8-fold
4	F	68	70	abdominal pain	tail	calcitonin	20.6-fold	15-fold
5	M	54	54	abdominal pain	tail	calcitonin	189-fold	NA
6	M	69	69	abdominal pain, weight loss	head	calcitonin	16.5-fold	2-fold
7	F	66	66	abdominal pain	tail	calcitonin	17.6-fold	not elevated
8	F	62	62	diarrhea, abdominal pain	tail	calcitonin	15-fold	2.7-fold
9	F	49	50	NME, wasting, anemia	tail	glucagon	46-fold	56-fold
10	M	66	66	NME, foot and leg exanthem, weight loss, diarrhea	head	glucagon	26.5-fold	1.2-fold
11	M	73	73	diarrhea, nausea, vomiting	head	glucagon	3-fold	2.6-fold
12	M	27	28	diabetes mellitus, weight loss	tail	glucagon	2.3-fold	not elevated

*F* female, *M* male, *Dx* Dignosis, *VIP* vasoactive intestinal peptide, *NME* necrolytic migratory erythema, *WDHA* watery diarrhea, hypokalemia, and achlorhydria, *NA* not available.

*associated with MEN 1.

^§^factor of upper limit of normal range.

The time between diagnosis and the first surgery varied between one and 137 months. Preoperative imaging revealed the rf-pNENs in all 12 patients (Table 2). All but one tumour had a size > 20mm. For the visualisation of the primary tumour, EUS and MRI were the most sensitive methods, with 100% detection and accuracy. Four patients underwent SRS until 2013, but after 2014, Ga68-DOTATOC PET/CT was performed on another five patients. SRS imaging showed the primary tumour in eight of nine patients, lymph node metastases in none of the patients, but liver metastases in all six patients with histologically confirmed distant metastases. It is of note, that only EUS and MRI detected a small (12 mm) glucagonoma in the pancreatic head. EUS-guided fine needle aspiration (FNA) was performed in four patients and confirmed the presence of pNENs in all four cases. The tumour was located in the pancreatic tail in nine patients and in the pancreatic head in three patients.

**Table 2:** Preoperative imaging.

	Preoperative imaging	US	EUS	CT	MRI	SRS	Ga68-DotatocPET/CT	IOUS
Number of patients	12	10/12	6/12	8/12	8/12	4/12	5/12	12/12
Primary tumor visualized	12/12	10/10	6/6	8/8	8/8	3/4	5/5	12/12
Lymph node metastases visualized	2/5	1/5	2/5	1/5	1/5	0/4	0/5	0/5
Distant metastases visualized	6/6	6/6	0/6	4/4	4/4	4/4	2/2	6/6

*US* ultrasonography, *EUS* endoscopic ultrasonography, *CT* computed tomography, *MRI* magnetic resonance imaging, *SRS* somatostatin receptor scintigraphy, *Ga68 DOTATOC-PET-CT* Gallium68 positron emission tomography, *IOUS* intraoperative ultrasonography.

Eleven of the 12 rf-pNENs were considered completely resectable based on preoperative imaging. Eight patients underwent distal splenopancreatectomies, one patient underwent pylorus preserving pancreaticoduodenectomy, one patient underwent total pancreatectomy, and one patient underwent enucleation of a 12mm glucagonoma in the pancreatic head. The remaining patient with a large VIPoma with far advanced diffuse metastatic disease in the liver and the peritoneum had only an exploratory laparotomy with palliative ileocecal resection for bowel obstruction without removal of the primary tumour. Ten patients underwent conventional open surgery, and the remaining two patients underwent robotic-assisted procedures. In addition to the pancreatic resection, five patients also underwent resection of liver metastases. In nine of the 12 patients, complete (R0) resection of the primary tumour and, if present, liver metastases was achieved, so that serum hormone levels dropped to normal levels postoperatively.

Postoperative complications (Clavien-Dindo > III) occurred in four patients (all three with VIPoma and one with glucagonoma), all of whom had to be reoperated. One of them was due to peritonitis, with a suspected anastomotic leak. However, this has not been confirmed. The second patient required open surgical abdominal lavage and drainage of a subphrenic and paracolic abscess. The third patient underwent metachronous splenectomy because of haemorrhage. The last patient underwent two reoperations because of tumour-associated perforation of the small intestine. He died on the 30th postoperative day after the initial surgery due to multi-organ failure. None of the patients developed postoperative pancreatic fistulas type B or C. The operations and postoperative complications of all patients are summarised in Table 3.

**Table 3:** Operations and postoperative complications.

Patient No	Tumour	Operation(s)	Postoperative complications (CD ≥ III)	Reoperation(s)
1	VIP 1	1. DPR, nephrectomy, splenectomy 2. Tumor resection, diaphragma resection cholecystectomy 3. Tumor resection, left nephrectomy 4. LR 5. LR 6. DPR, CR, SR 7. Explorative laparotomy only	after 1. surgery—IIIb- abdominal abscess	laparotomy, lavage, drainage
2	VIP 2	Palliative ileocecal resection, LR	IIIb—peritonitis	laparotomy, lavage
3	VIP 3	DPR, CR, splenectomy, LR	IIIb intestinal perforation, later V	2 × laparotomy, resection of small intestine
4	CT 1	DPR, GR, splenectomy, cholecystectomy, LR	No	No
5	CT 2	DPR, splenectomy	No	No
6	CT 3	TP, splenectomy, cholecystectomy	No	No
7	CT 4	DPR, splenectomy	No	No
8	CT 5	1. DPR, splenectomy, cholecystectomy, LR 2. Lymphadenectomy	No	No
9	Glu 1	DPR, splenectomy, cholecystectomy, lymphadenectomy, LR	No	No
10	Glu 2	PPPD, LR	No	No
11	Glu 3	1. Enucleation 2. PPPD, cholecystectomy	No	No
12	Glu 4	DPR	IIIb—Bleeding	laparotomy, splenectomy

*VIP* VIPoma, *CT* calcitoninoma, *Glu* glukagonoma, *DPR* distal pancreatic resection, *TP* total splenopancreatectomy, *PPPD* pylorus-preserving pancreatic head resection, *CR* colon resection, *SI* small intestine, *GR* gastric resection, *LR* liver resection.

Rf-pNENs were confirmed by histopathological examination in all patients. All 12 primary tumours were immunohistochemically positive for chromogranin A, synaptophysin, and their respective secreted hormones. All primary tumours were well differentiated and graded G1 in three patients and G2 in the remaining nine patients. The tumour was classified as T1 in three patients, T2 in three, T3 in three, and T4 in two patients. Six patients had locoregionally limited, Stage I-III rf-pNENs, and the other six patients had Stage IV disease. The median tumour size was 82 (range 12–220) mm. Five of the 12 patients had lymph node metastasis. A median of 13 (range 5–28) lymph nodes were removed. In nine of the 12 patients, resection was defined as R0 resection. Histopathology results and hormone levels after initial surgery are summarised in Table 4.

**Table 4:** Histopathology and hormon levels after initial surgery.

Patient No	Tumour	TNM	UICC	R	Ki-67 Index (%)	Tumour Grade	Tumour size (mm)	Post-OP cure of symptoms	Post-OP normalized hormone level	Post-OP serum hormone level (x-fold)^§^
1	VIP 1	T4N1(8/24)M0	III	R0	1	1	220	Yes	Yes	0.2
2	VIP 2	TxNxM1(HEP)	IV	R2	1	1	35	No	No	1.6
3	VIP 3	T4N1(3/12)M1 (HEP)	IV	R0	10	2	80	No	No	2.2
4	CT 1	T2N0(0/6)M1 (HEP)	IV	R1	5	2	40	Yes	Yes	0.7
5	CT 2	T3N1(7/22)M0	III	R0	5	2	130	Yes	Yes	0.4
6	CT 3	T2N0(0/22)M0	II	R0	6	2	75	Yes	Yes	0.4
7	CT 4	T2N0(0/16)M0	II	R0	4	2	35	Yes	Yes	0.2
8	CT 5	T3N1(1/10)M1 (HEP)	IV	R1	3	2	113	Yes	Yes	< 0.1
9	Glu 1	T1N1(1/15)M1 (HEP)	IV	R0	10	2	45	Yes	No	4.4
10	Glu 2	T3N1(1/17)M1 (HEP)	IV	R0	5	2	85	Yes	Yes	0.8
11	Glu 3	1.T1NXM0 2. T1N0(0/17)M0	I	R0	2	1	12 11	Yes	Yes	0.2
12	Glu 4	T1N0(0/17)M0	I	R0	3	2	117	Yes	Yes	< 0.1

*VIP* Vipoma, *CT* calcitoninoma, *Glu* glucagonoma, *HEP* liver.

^§^factor of upper limit of normal range.

## Follow-up

**Figure 1:**
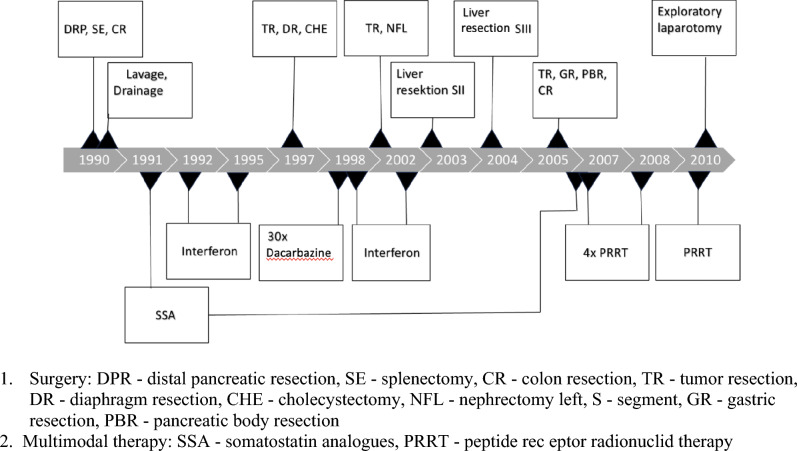
Course of a VIPoma patient over 20 years.

The median follow-up period was 75 months (range 1–247 months). Four of the six patients with resected localised disease (stages I-III) remained disease-free for a median of 61 (range 6–108) months. In this group, one patient experienced local recurrence six months after glucagonoma enucleation from the pancreatic head, which required subsequent pylorus-preserving pancreatic head resection. Following this reoperation, the patient remained disease-free until the last follow up 90 months after surgery. Another patient in this group had local tumor recurrence 84 months after resection of a 22 cm-sized initially locoregionally metastasised VIPoma in the pancreatic tail. This patient underwent a total of seven redo-surgeries and more than 40 cycles of multimodal therapy (SSA, interferon, PRRT, chemotherapy) due to tumour recurrence and/or progression. All the treatment modalities are summarised in Fig. 1. The sequence of multimodal treatment led to a survival of 247 months after initial diagnosis.

In the six patients with stage IV disease, multimodal treatments were performed, including somatostatin analogues, chemotherapy regimens with dacarbazine, doxorubicin, streptozotocin, capecitabine, targeted therapy with sunitinib, radiofrequency ablation or TACE of liver metastases, recurrent PRRTs, and reoperations. One patient with a Stage IV calcitoninoma developed a local lymph node recurrence 11 months after the initial surgery, and excision of this metastasis was performed. He was alive with the disease after 49 months of follow-up. The remaining five patients died of disease 1, 20, 48, 52, 114, and 247 months after the initial surgery. See Table 5.

**Table 5:** Follow-up.

Patient No	Tumour	Follow-up after first surgery (months)	Recurrence of disease (months)	Development of distant metastases	Disease status
1	VIP 1	247	84	Hep, P, KL	DOD
2	VIP 2	52	persistence	persistence	DOD
3	VIP 3	1	No	No	DOD
4	CT 1	48	9	Hep	DOD
5	CT 2	108	No	No	NED
6	CT 3	92	No	No	NED
7	CT 4	50	No	No	NED
8	CT 5	49	11	Hep, LN	AWD
9	Glu 1	20	3	Hep, LN	DOD
10	Glu 2	114	3	Hep	DOD
11	Glu 3	96	6	No	NED
12	Glu 4	26	No	No	NED

*VIP* Vipoma, *CT* calcitoninoma, *Glu* glukagonoma, *Hep* hepar, *LN* lymph node, *P* peritoneum, *KL* kidney left, *NED* no evidence of disease, *AWD* alive with disease, *DOD* death of disease.

Among five patients who experienced recurrence after initially normalised hormone levels, three patients had biochemical evidence of disease synchronously with tumour recurrence on imaging, the remaining patients developed tumours on imaging three, 14 and 36 months after evidence of increased hormone levels.

One-year survival in our cohort was 91%, and the true five-year survival rate was 55%. In three patients follow-up within this study was less than five years.

The characteristics, treatments, and follow-up of the individual tumour subtypes are summarised in Table 6.

**Table 6:** Characteristics of the patients with regard to entity.

	Rf-pNEN(n = 12)	CT-pNEN (n = 5)	Glucagonoma (n = 4)	VIPoma (n = 3)
Median age at initial surgery (range)	60 (28–73)	64 (54–70)	54 (28–73)	58 (32–73)
Sex (male/female)	7/5	2/3	3/1	2/1
Presence of MEN1 or VHL	1/12	0/5	0/4	1/3
Tumour size, mm, median (range)	82 (12–220)	79 (35–130)	65 (12–117)	112 (35–220)
Tumour location (PH/PB/ PT)	3/0/9	1/0/4	2/0/2	0/0/3
Ki 67% median (range)	5 (1–10)	5 (3–6)	5 (2–10)	4 (1–10)
Stage I/II/III/IV	2/2/2/6	0/2/1/2	2/0/0/2	0/0/1/2
Surgical resection primary tumour	11/12	5/5	4/4	2/3
Intended curative resection	9/12	3/5	4/4	2/3
Additional treatment	6/12 (50%)	2/5	2/4	2/3
Somatostatin analogues	6/12	2/5	2/4	2/3
Chemotherapy	3/12	0/5	1/4	2/3
Sunitinib	1/12	0/5	1/4	0/3
PRRT	2/12	0/5	1/4	1/4
TAE/TACE	1/12	1/5	0/5	0/5
Radiofrequency ablation	1/12	1/5	0/5	0/5
Recurrence after curative resection, number (%)	5/12 (42%)	1/5	3/4	1/1
Tumour related death, n (%)	6/12 (50%)	1/5	2/4	3/3
5-years survival (%)	55%	50%	50%	33%
Follow- up (months)	73	69	64	100

*rf-pNEN* rare functional pancreatic neuroendocrine neoplasms, *VIP* Vipoma, *CT* calcitoninoma, *Glu* glucagonoma, *PH* pancreatic head, *PB* pancreatic body, *PT* pancreatic tail, *PRRT* peptide receptor radionuclide therapy, *RFA* radiofrequency ablation, *TAE/TACE* transarterial embolisation/transarterial chemoembolization.

## Discussion

In our series, patients with rf-pNENs had a median age of 61.5 (range 28–73 years), which is in the range reported in the literature^7,14,22,32,33^. In addition, no sex predominance was noted in either our series or in the literature. All three patients with VIPoma presented with the classic symptomatology of watery diarrhoea, which is typical in these patients^11,12,32^. However, only two of four patients with glucagonoma in the present series had necrolytic migratory erythema, and only one patient had diabetes. This is in line with a previous Swedish study of 23 patients^22^. Only 22% of these patients had developed diabetes before the diagnosis of glucagonoma, and necrolytic migratory erythema was diagnosed in only 52% of patients. In a recent clinicopathological study of 25 pNENs with calcitonin expression in immunohistochemistry, none of the patients had symptoms of calcitonin excess, eight had an insulinoma and one patient had symptoms of a somatostatinoma^14^. Elevated serum calcitonin levels were not reported in the clinical records of any of these 25 cases. The five patients presented here all had significantly elevated calcitonin serum levels up to 150-fold. However, only the patient with a 150-fold elevated calcitonin had diarrhoea, which is typical for excessive hypercalcitonemia. Abdominal pain due to tumour size was a leading symptom in three of our 12 (25%) patients with rf-pNEN, which was reported to be present in 19.8%-33% of other case series^7,10,34,35^.

In the present cohort, there was a predominant tumour location in the left pancreas (75%), which is in line with previous case series of VIPomas, glucagonomas, and calcitonin producing pNENs^7,14,22,32,33^. The median diameter in our cohort was 82 (range 12–220), and only one patient with a glucagonoma had a tumour size < 20mm. In an older literature review of 407 patients with glucagonoma, 80 (29%) patients had small tumours of 20mm or less and 8.8% of these patients had metastases^7^. The median tumour size in the present cohort was larger than that previously reported for VIPoma, with a median diameter of 32–57.5 mm^33^, glucagonoma with a median size from 50 to 55 mm^22,36^, and calcitonin producing pNENs with a median size of 48 mm^14^.

As in other studies, patients with rf-pNENs frequently show liver metastases at the time of initial diagnosis as in 50% of patients in our cohort. This is in the range of reported rates for VIPoma of 36–75%^32–34,37^ and glucagonoma of 50–78%^8,22,36^. It is of note, that in the present series of patients who underwent surgery, two of five calcitonin producing pNENs presented with liver metastases, whereas in a previous series only four of 23 (17%) tumours did so^14^. None of our patients with rf-pNENs showed initial bone metastases, whereas a rate of 6–15% has been reported previously^7,8,32^. This might in part be due to the lower sensitivity of SRS scintigraphy imaging used in the early 2000s.

All 12 rf-pNENs in the present cohort were G1 (n = 3) or low G2 (n = 9) tumours with a Ki67 index in the primary tumour of at most 10%. This is in line with the Ki67 index in the majority of reported glucogonomas^22,36^ and calcitonin producing pNENs^14^. For VIPoma, however, G3 tumours have been described in up to 27% of cases^37^.

In the present cohort, all rf-pNENs could be visualised by preoperative imaging with either CT or MRI because of the large tumour size, as reported in the previous series^37–40^. EUS was used in only half of our patients and had an additional value in only one patient with 12 mm glucagonoma in the pancreatic head. According to recent guidelines^3,5^, imaging with 68Ga-labelled somatostatin analogues with PET/CT is more sensitive and highly specific for rare pNENs and is therefore recommended as the first-line diagnostic imaging method for staging in patients with rf-pNENs. Our cohort supports this recommendation since eight of nine patients who underwent SRS imaging showed positive primary tumours and in all five metastatic patients who underwent SRS imaging, metastasis could be visualised. In patients with rapid tumour growth and/or high G2 or G3 tumours, 18-FDG-PET/CT can also be considered to assess tumour burden^41,42^. In our cohort, this was not applied because we had only G1 and low G2 tumours.

The indications for surgery are influenced by clinical symptom control, the technical possibility of local R0 resection, and the presence and extent of metastatic spread^3,5,43^. Curative intended surgery should always be indicated, even in the presence of metastatic disease, if a complete resection can be achieved and the patient is fit for surgery^5,34^. In the present cohort, we could achieve a potential curative R0 resection, documented by postoperative normal serum hormone levels, in nine of 12 (75%) patients of whom three also had liver metastases. The surgery rate as well as the R0 resection rate are comparable to other series, ranging from 28 to 63%^11,34,37,39,44^. In two patients with calcitonin producing pNENs with liver metastases, R0 resection was attempted based on preoperative imaging, but only an R1 resection could be achieved. However, this fact is not considered to indicate failure since elevated hormone levels dropped to the normal range postoperatively. In addition, according to ENETS guidelines, debulking surgery can be considered in rf-pNEN if at least 80% of the gross tumour is thought to be resectable^5,43^. The type of surgery for rf-pNENs depends on the location of the primary tumour. Because of the usually large rf-pNEN size and high prevalence of liver metastases, curative surgery usually requires formal pancreatic resection with lymphadenectomy^2,5,43^. This was performed in 10 of 11 patients in the present series, as well as in the majority of patients in other series^11,37^. Even for small rf-pNEN, parenchyma-sparing resection, such as enucleation, might be insufficient. One of our patients had a robotic-assisted enucleation of 12 mm-sized glucagonoma out of the pancreatic head with negative lymph node sampling and developed local recurrence six months later. Consequently, the patient underwent partial pancreaticoduodenectomy, which has resulted in a disease-free survival of 90 months so far.

Five of the six patients with recurrent disease and one patient with an unresectable VIPoma underwent several cycles of multimodal treatment, including redo surgery, somatostatin analogues, chemotherapy, targeted therapy with sunitinib, local ablative therapies with TACE and/or radiofrequency ablation and PRRT (see Table 5). Although more therapies have become available for pNENs during the last decade, including targeted therapies with e.g. sunitinib, new chemotherapy regimens (e–g. Tem/Cap), and PRRT, no significant data has yet been compiled on the oncological outcomes in patients with rf-pNEN. Thus, the discussion of the possible advantages and disadvantages of individual treatments and their sequence is somewhat vague and is beyond the scope of this article. However, somatostatin analogues were used as the mainstay treatment in several other series^11,32,37,40^ when curative surgery was not an option. They can control symptoms caused by excessive hormone secretion and prolong progression-free survival^45^. This was the case in six of seven patients with recurrent or persistent disease in our cohort. Since the majority of rf-pNEN somatostatin receptor-positive grade 1/2 tumours, 8 of 9 investigated in the presented series, PRRT is currently a very good option to control symptoms of hormonal excess as well as tumour growth progression^11,46,47^. In a previous series, PRRT was the most efficacious second-line treatment in patients with VIPoma who had refractory WDHA syndrome despite receiving the maximum doses of SSA^32^. It is also noteworthy that repeated surgery for disease recurrence should always be considered in the multimodal concept. One of our patients with a 22cm sized VIPoma underwent—embedded in SSA treatment, chemotherapy, and PRRT—a total of seven reoperations, which resulted in a survival of over 20 years, as summarised in Fig. 1.

Since the treatment armamentarium is complex, all patients with advanced rf-pNENs should be discussed in a multidisciplinary tumour board, ideally in centres of excellence.

In the present study, the eight patients with potentially curative surgery had a five-year survival of 63%, which is similar to that reported by others^8,34,44,48,49^.

The study from Azizian et al. showed that patients who underwent surgery had a longer overall survival than patients who were treated with other therapeutic modalities (44 vs. 33 months)^11^. Five (42%) patients remained free of disease for a median of 74 months after initial surgery. This outcome is similar to that of previous studies of rf-pNENs ranging from 15 to 180 months^39,44,50,51^.

A study by Sakurai et al. reported a disease-free interval after an initial surgery of 180 months. After a 180-month disease-free interval, this patient underwent a second curative surgery for the locally recurrent VIPoma. The patient is alive with no relapse 14 months after the second surgery^51^. These cases demonstrate the importance of long-term observation of patients.

The study from Murakami et al. showed, that the median survival time for patients with VIPoma was 71 months^33^. In the present study, the three patients with VIPoma showed a five-year tumour survival of 33% and a median survival time of 100 months.

It is crucial to distinguish between cases in which all metastases and the primary tumour are resectable and cases in which complete resection of all lesions is not possible (debulking surgery). The survival of the palliatively operated three patients in the present study was 48, 49, and 52 months. Brugel et al. showed in a retrospective analysis of four cases, a debulking surgery with palliative intent. The median progression-free survival was 21 months^34^.

It should be mentioned that calcitonin producing pNENs might be less aggressive than VIPoma or glucagonoma since according to a recent study of 25 calcitonin producing pNENs, 60% of patients were alive with no evidence of disease and 20% survived with disease after five years of follow-up^14^. This tendency can be confirmed by the present study.

The present study has three major limitations. First, the collective size is very small, as is usual for these very rare tumour entities. Second, the retrospective design implies bias and missing data. _ Considering the large variety of therapeutic options and the small number of patients, prospective registration in international databases (e.g., the ENETS-Database) is required to better understand the characteristics and outcomes of these extremely rare tumours.

## Conclusion

In conclusion, rf-pNENs are a heterogeneous group of tumours with a good long-term prognosis, if initially radically resected. Long-term survival can even be achieved in metastasised tumours using multimodal treatment. As these tumours are extremely rare, treatment in expert centres is recommended. Through a comprehensive analysis of these extremely rare tumours, valuable insights into the management of these tumours are provided.

## Data Availability

The data that support the findings of this study are not publicly available because they contain information that could compromise the privacy of research participants but are available from one of the authors [M.S.] upon reasonable request.
